# Nonlinear ABAQUS Simulations for Notched Concrete Beams

**DOI:** 10.3390/ma14237349

**Published:** 2021-11-30

**Authors:** Ahmed Bahgat Tawfik, Sameh Youssef Mahfouz, Salah El-Din Fahmy Taher

**Affiliations:** 1 Construction and Building Engineering Department, College of Engineering and Technology, Arab Academy for Science, Technology and Maritime Transport (AASTMT), B 2401 Smart Village, Giza 12577, Egypt; symahfouz@aast.edu; 2 Professor of Concrete Structures, Structural Engineering Department, Tanta University, Tanta 31527, Egypt; salah.taher@f-eng.tanta.edu.eg

**Keywords:** ABAQUS, finite element analysis (FEA), concrete damage plasticity (CDP), extended finite element method (XFEM), external post-tensioning (EPT)

## Abstract

The numerical simulation of concrete fracture is difficult because of the brittle, inelastic-nonlinear nature of concrete. In this study, notched plain and reinforced concrete beams were investigated numerically to study their flexural response using different crack simulation techniques in ABAQUS. The flexural response was expressed by hardening and softening regime, flexural capacity, failure ductility, damage initiation and propagation, fracture energy, crack path, and crack mouth opening displacement. The employed techniques were the contour integral technique (CIT), the extended finite element method (XFEM), and the virtual crack closure technique (VCCT). A parametric study regarding the initial notch-to-depth ratio (a_o_/D), the shear span-to-depth ratio (S.S/D), and external post-tensioning (EPT) were investigated. It was found that both XFEM and VCCT produced better results, but XFEM had better flexural simulation. Contrarily, the CIT models failed to express the softening behavior and to capture the crack path. Furthermore, the flexural capacity was increased after reducing the (a_o_/D) and after decreasing the S.S/D. Additionally, using EPT increased the flexural capacity, showed the ductile flexural response, and reduced the flexural softening. Moreover, using reinforcement led to more ductile behavior, controlled damage propagation, and a dramatic increase in the flexural capacity. Furthermore, CIT showed reliable results for reinforced concrete beams, unlike plain concrete beams.

## 1. Introduction

Employing numerical finite element (FE) simulations for different concrete elements reduces the need for further physical testing and helps researchers undertake complex parametric studies precisely. The complex nonlinear behavior of concrete in both tension and compression makes it challenging to simulate. The complexity stems from the brittle response of concrete, leading to failure due to cracking or crushing when subjected to tension or compression, respectively. Also, it is difficult to simulate crack initiation and propagation due to the tensile damage.

Various constitutive numerical models were presented to simulate the concrete behavior in tension and compression [1,2,3,4,5]. Y. Nikaido [6] improved a constitutive numerical model to simulate concrete behavior by considering compression stiffness recovery. Furthermore, different studies were conducted to simulate the nonlinear behavior of different plain and reinforced concrete elements [7,8,9,10,11]. Zhang et al. [12] compared different numerical crack simulation techniques to simulate a notched concrete beam using the unified FE software package ABAQUS [13] without experimental result validation. These techniques included the virtual crack closure technique (VCCT) and the extended finite element method (XFEM). It was deduced that both VCCT and XFEM can capture the softening regime for concrete fracture well. An experimental work achieved by Yin et al. [14] was conducted on notched plain concrete beams having different spans. The experimental scope was set to determine the effect of changing the span on different concrete fracture parameters.

Many studies integrated the use of different composite materials with notched concrete beams through various experiments [15,16,17,18]. This integration aimed to enhance the flexural performance and to investigate the fracture and bond behavior of concrete beams. Zhong et al. [15] experimentally investigated the effect of installing a channel steel plate ahead of a concrete beam notch on the strengthening performance. It was found that placing a channel steel plate at the notch tip of the concrete beam can significantly increase the load carrying capability against mode 1 fractures. Additionally, an increase in fracture energy and ductile softening were captured after placement. Furthermore, the fracture failure of the tested beams was significantly influenced by the debonding and slippage behavior between the steel plate channel and the concrete beam. It was noted that steel plate size has no significant effect on flexural capacity. Sun et al. [16] studied the effect of using different volumetric dosages of basalt fibers on the fracture resistance of precast notched concrete beams. It was deduced that the peak load, the initiation toughness, and the fracture energy significantly increased after increasing the basalt fiber dosage. Moreover, the fracture energy and the unstable toughness had no size effect, unlike the initiation toughness which increased with increased specimen height.

De Domenico et al. [17] investigated the interfacial characteristics of the carbon fiber-reinforced polymers (CFRP) system and the fiber-reinforced cementitious matrix with polybenzoxole and cement-based mortar (PBO-FRCM) system. Both systems were adhesively bonded at the bottom of the notched concrete beams to study the effect of environmental conditions on the bond and the ultimate capacity for each system. It was found that the PBO-FRCM system was not affected by environmental conditions. Conversely, the CFRP system was affected by the curing conditions, as more than a 30% reduction in the average peak load was noticed compared to non-conditioned beams.

Chen et al. [18] conducted three-point bending tests on notched steel fiber-reinforced concrete (SFRC) beams containing single or novel multiple hooked-end steel fibers to study the effects of fiber aspect ratio, fiber length, and fiber shape on flexural tensile strength. It was concluded that the limit of proportionality, which is related to the initial concrete cracking, is mainly dependent on the concrete strength, and not on the fiber properties. On the other hand, it was found that increasing the fiber dosage, length, aspect ratio, and number of hooked-ends enhanced the residual flexural tensile strength in the post-cracking stage.

The previously mentioned studies focused on the incorporation of different composite materials with notched concrete beams to enhance flexural tensile capacity and to improve cracking damage resistance. However, studies scrutinizing the effects of geometrical characteristics on the flexural performance of notched concrete beams are lacking. Along this research line, this paper provides numerical investigations regarding the effects of different geometrical aspects, such as loading scheme and notch height, on flexural performance. Additionally, this paper provides further numerical investigations on methods of enhancing the flexural tensile capacity and damage control performance of notched concrete beams. Unlike the studies mentioned in the literature that addressed this problem using different composite materials, the current study implemented steel reinforcement and un-bonded post-tensioning. Moreover, the simulations conducted in the current work were achieved by employing different numerical techniques in a comparative study to determine the most appropriate technique for concrete fracture simulation. 

The current study utilizes the experimental work conducted by Yin et al. [14]. The current work aims to study the flexural behavior and fracture initiation and propagation of notched plain and reinforced concrete beams. Additionally, different parametric studies were conducted to understand the influence of changing different parameters on the flexural response. The studied parameters were the initial notch-to-depth ratio (a_o_/D), the shear span-to-depth ratio (S.S/D), and external post-tensioning (EPT). The flexural behavior, damage initiation and propagation of six notched plain concrete beams having various span-to-depth ratios (S/D) were numerically investigated. All investigated beams were subjected to a three-point bending setup. Their response was captured as a relationship between loading level (P) and crack mouth opening displacement (CMOD). Three techniques for crack modeling in ABAQUS, such as the contour integral technique (CIT), XFEM and VCCT were considered, to determine the most suitable crack modeling technique. These techniques employed different material damage criteria: concrete damage plasticity (CDP), maximum principal stress (MAXPS) and critical energy release rate (G_c_). The numerical results were compared with those of the experimental investigation to find out the most suitable technique for expressing the behavior and the damage of all tested beams.

Section 2 of this paper illustrates the details of the experimental works and reviews their outcomes. Section 3 discusses the details of developing the numerical FE models. Section 4 presents the numerical simulation outcomes. Section 5 illustrates the parametric studies conducted, their results, and discussion. Section 6 provides a summary of the obtained conclusions.

## 2. Synopsis of Experimental Data

**Figure 1:**
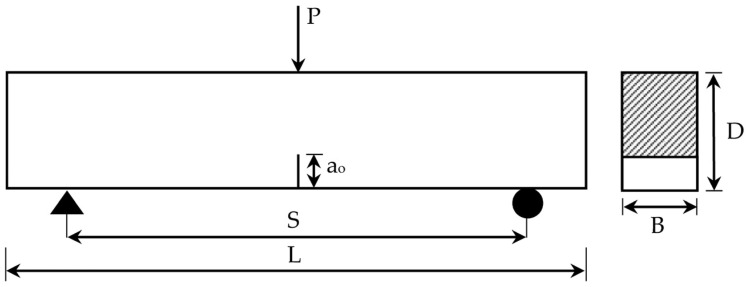
Experimental test loading scheme used.

According to the experimental work done by Yin et al. [14], a three-point-bending test was carried out, as indicated in Figure 1. This test was conducted on six series of notched plain concrete beams having different S/D ratios. Each beam series included four typical beams. The depth (D) and the breadth (B) of each beam were 150 mm and 100 mm, respectively. The length (L) of beams ranged from 375 mm to 1100 mm, while the corresponding span (S) ranged from 300 mm to 900 mm. These span ranges were selected to achieve S/D ratios ranging from 2 to 6. An initial notch (a_o_) of 60 mm at the middle of all beams was considered. Table 1 illustrates the details of beams. The behavior of each beam was captured in the form of a P-CMOD relationship. These relationships, along with the crack propagation path after cracking, are also provided.

## 3. Evolution of the Numerical FE Models

This section explains the details of developing ABAQUS FE models employing CIT, VCCT and XFEM. 

### 3.1. Contour Integral Technique

The contour integral technique (CIT) was used to study the commencement of damage in quasi-static problems [13]. To use CIT, the crack front region, crack line, and extension direction were specified. Moreover, a region with separate faces was modeled to be free to move apart as the crack mouth began to open. These faces were created using the seam command in ABAQUS. To simulate the nonlinearity of concrete, the CDP model was employed. The CDP model was utilized for its capability of expressing the inelastic responses of concrete and quasi-brittle materials, as well as damage characteristics in both compression and tension.

#### 3.1.1. Concrete Tensile Behavior

The uniaxial behavior of concrete in tension was defined using a relationship between the tensile stresses and their corresponding cracking strains. Defining this relationship makes it necessary to convert the tensile strain ($\varepsilon_{t}$) to the cracking strain ($\varepsilon_{t}^{\sim ck}$) using Equation (1).

(1) $$\varepsilon_{t}^{\sim ck}=\varepsilon_{t}-\varepsilon_{ot}^{el}$$

where $\varepsilon_{ot}^{el}$ refers to the tensile elastic strain of the undamaged material condition and equals $\sigma_{t0}$*/*$E_{o}$*;* $\sigma_{t0}$ is the maximum elastic tensile stress and $E_{o}$ is the concrete modulus of elasticity.

A modified tension stiffening material model suggested by Wahalathantri et al. [19] was used in the present work to obtain the uniaxial tensile stress–strain relationship. The modifications were made to overcome convergence problems and to avoid ABAQUS solution errors. This model is based on the tension stiffening model of Nayal and Rasheed [20].

#### 3.1.2. Concrete Compressive Behavior

The uniaxial behavior of concrete in compression was defined using a relationship between the compressive stresses and their corresponding inelastic strains. Based on the ABAQUS documentation, the compressive strain ($\varepsilon_{c}$) was converted to the inelastic strain ($\varepsilon_{c}^{\sim in}$) to define this relationship by employing Equation (2).

(2) $$\varepsilon_{c}^{\sim in}=\varepsilon_{c}-\varepsilon_{oc}^{el}$$

where $\varepsilon_{oc}^{el}$ refers to the compressive elastic strain condition of the undamaged material and equals $\sigma_{c}$*/*$E_{o}$, and $\sigma_{c}$ is the maximum elastic compressive stress.

A numerical material model for concrete developed by Hsu et al. [21] was employed to obtain the stress–strain relationship in compression. This material model can be used for concrete material with a concrete cube compressive strength ($\sigma_{cu}$) up to 62 MPa [19,21]. It considers that the stress–strain relationship behaves linearly up to 50% of $\sigma_{cu}$ in the hardening portion of the relationship. Afterward, numerical material model equations were used to describe the relationship until the softening occurred and reached the value of 30% of the $\sigma_{cu}$ in the softening portion. The numerical material model equations can be expressed as follows:

(3) $$\sigma_{comp.}=\left(\frac{\beta \left(\varepsilon_{c}/\varepsilon_{o}\right)}{\beta -1+{\left(\varepsilon_{c}/\varepsilon_{o}\right)}^{\beta }}\right)\sigma_{cu}$$

(4) $$\beta =\frac{1}{1-\left[\frac{\sigma_{cu}}{\varepsilon_{o}E_{o}}\right]}$$

(5) $$\varepsilon_{o}=8.9\times 10^{-5}{\;\sigma }_{cu}+2.114\times 10^{-3}$$

(6) $$E_{o}=1.2431\times 10^{2}\;\sigma_{cu}+3.28312\times 10^{3}$$

where $\sigma_{comp.}$ is the compressive stress. The parameter β is a parameter that depends on the stress–strain diagram shape, and $\varepsilon_{o}$ is the compressive strain at peak stress.

#### 3.1.3. Concrete Damage Parameters

The concrete damage plasticity (CDP) model expresses concrete damage utilizing a damage parameter ranging between zero (indicating the intact material state) and one (indicating a complete loss of material strength). Thus, the operative modulus of elasticity after tensile and compressive damage can be evaluated using Equations (7) and (8), respectively.

(7) $$E_{t}=E_{o}\times \left(1-d_{t}\right)$$

(8) $$E_{c}=E_{o}\times \left(1-d_{c}\right)$$

where $d_{t}$ and $d_{c}$ refer to the tensile and compressive damage parameters, respectively, and can be evaluated using Equations (9) and (10), respectively.

(9) $$d_{t}=1-\left(\frac{\sigma_{t\;}}{\sigma_{t}^{\prime }}\right)$$

(10) $$d_{c}=1-\left(\frac{\sigma_{c\;}}{\sigma_{c}^{\prime }}\right)$$

where $\sigma_{c}^{\prime }$ and $\sigma_{t}^{\prime }$ are the effective compressive and tensile strength, respectively. 

Along with stress–strain relationship and damage parameter data, five plasticity parameters are needed to define the CDP model in ABAQUS. These parameters can be described as follows:

1. The dilation angle (*Ψ*) measured in the p–q plane at high confining pressure and is necessary to evaluate the potential plastic flow, which uses the Drucker–Prager hyperbolic function. The dilation angle ranges between $0^{{}^{\circ}}$ to $\;56^{{}^{\circ}}$ [5]. In the current research, a value of $31^{{}^{\circ}}$ was used, according to Hafezolghorani et al. [22].
2. Flow potential eccentricity (*ϵ*)designates the rate at which the hyperbolic Drucker–Prager function reaches the linear Drucker–Prager function. A value of 0.1 was used. This value guarantees that the material has nearly the same dilation angle among a wide band of confining pressure values [13].
3. The ratio of initial equibiaxial compressive strength (*f_b0_*) to the initial uniaxial compressive strength (*f_c0_*) is responsible for the evolution of yield surfaces. This ratio contributes to the evaluation of the yield function proposed by Lubliner et al. [23] and modified by Lee and Fenves [24]. This parameter ranges between 1.10 and 1.16 [13]. The default value of 1.16 is used by many researchers [25] and was adopted in the current study.
4. The ratio (*K_c_*) of the second stress invariant on the tensile meridian ($q_{TM}$) to that on the compressive meridian ($q_{CM}$) contributes to evaluating the yield function. It ranges between 0.5 and 1 [26]. The default value of 2/3 was employed in the present simulation as per many researchers [25].
5. The viscosity parameter (*μ*) helps to make the tangent stiffness of the degrading material have a positive value for small time increments. This value is achieved by allowing stresses to be outside the developed yield surfaces. Using small values of µ compared to the characteristic analysis time increment tends to enhance the rate of solution convergence in the softening regime. The default value of zero [13] caused premature termination of the analysis due to the damage that occurred in the element. According to Tao et al. [5], *μ* has no significant effect on the analysis precision. Thus, a value of 0.0007 was utilized.

In the current research, a reduced integration quadratic 20-node brick element (C3D20R) was used. This element is a second-order element type, which gives higher accuracy and is effective in bending dominated problems [13]. As result of a sensitivity study for mesh size, a fine mesh size of 10 mm was used for the different models made for the present study.

### 3.2. Virtual Crack Closure Technique

**Figure 2:**
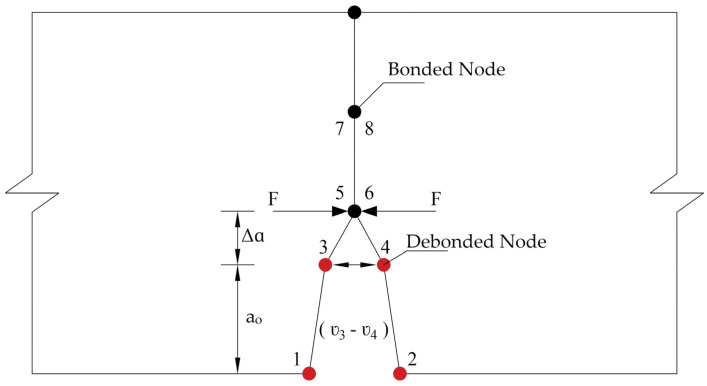
Virtual crack closure technique (VCCT) debonding at crack location.

The virtual crack closure technique (VCCT) employs the principles of linear elastic fracture mechanics (LEFM) [13,27] and Irwin’s criterion [28]. Rybicki and Kanninen [29] presented this technique, and it was improved by Raju [30] as higher-order interpolation elements were added. This technique assumes that once the crack is opened to a specific extent, it releases the same amount of strain energy needed to close the crack by the same extent [13]. As shown in Figure 2, nodes 1, 2, 3, and 4 were debonded nodes, while the others were bonded nodes. The energy release rate of mode I fractures $\left(G_{I}\right)$ for 4-noded elements can be estimated using Equation (11).

(11) $$G_{I}=\underset{\Delta a\to 0}{lim}\frac{1}{2b\Delta a}F_{5,6}(v_{3}-v_{4})$$

where Δa and b are the length and the width of the elements at the crack front, respectively. The symbol $F_{5,6}$ denotes the force between nodes 5 and 6. The displacements of nodes 3 and 4 are $v_{3}$ and $v_{4}$, respectively.

The VCCT technique requires a predefined crack path along which to propagate [31]. To define the crack path, the beam was modeled as two separate identical parts. A contact interaction was assigned to the bonded mutual nodes of the two parts. This interaction included a fracture criterion factor (f) that depends on the overall rate of energy release ($G_{T}$) and is fulfilled when the critical energy value ($G_{T}^{C}$) is achieved. The fracture criterion factor (f) is evaluated using Equation (12).

(12) $$f=\frac{G_{T}}{G_{T}^{C}}$$

A value of 0.06 N/mm was adopted for $G_{T}^{C}$ [14]. Based on the mesh sensitivity study, a fine mesh of reduced integration continuum 8-node biquadratic plane stress elements (CPS8R) was used for the VCCT models.

### 3.3. Extended Finite Element Method Technique

The extended finite element method (XFEM) utilizes the principles of LEFM until the crack is initiated. This technique was first proposed by Belytschko and Black [32], and was modified by Moës et al. [33]. The XFEM technique facilitates the study of crack propagation along an arbitrary path that depends on the solution. In this technique, the separation is performed by providing supplementary freedom degrees to elements around the crack path and the crack tip. It uses the partition of the unity property FE method of Melenk and Babuška [34], in which the summation of all shape functions equals one. The displacement function expressed in Equation (13) allows the propagation of the crack through the meshed elements without the need for remeshing [35].

(13) $$u=\overset{S_{I}}{\underset{I=1}{\sum }}N_{I}\left(x\right)u_{I}+\overset{S_{c}}{\underset{c=1}{\sum }}N_{c}\left(x\right)H\left(x\right)a_{c}+\overset{S_{t}}{\underset{t=1}{\sum }}N_{t}\left(x\right)\overset{4}{\underset{\alpha =1}{\sum }}F_{\alpha }\left(r,\theta \right)b_{t}^{\alpha }$$

where $S_{I}$ expresses the node count in the elements that contain the fracture, $S_{c}$ represents the node count within the elements that include the fracture line, and $S_{t}$, is the node count in the elements enclosing the fracture tip. The node shape functions for elements enclosing the crack tip, elements including crack line and elements containing a fracture are denoted by the symbols $N_{t}$, $N_{c}$ and $N_{I}$, respectively. The standard displacement of node I is denoted by $u_{I}$. Both $a_{c}$ and $b_{t}^{\alpha }$ are the coefficients that express the degrees of nodal enhanced freedom for the nodes related to the elements that enclose the crack line and the tip, respectively. H(x) is the Heaviside function across the crack surfaces and $F_{\alpha }\left(r,\theta \right)$ is the crack tip asymptotic enrichment function.

To initiate a discontinuity among the damaged elements to represent a crack, the Heaviside function H(x) is employed as expressed in Equation (14).

(14) $$H\left(x\right)=\{\begin{array}{cc} 1 & \;\mathrm{if}\;\left(x-x^{*}\right)\cdot n\geq 0\\ -1 & \;\mathrm{otherwise}\; \end{array}$$

where x is a sample (Gauss) point, $x^{*}$ is the point on the crack closest to x and n is the unit outward normal to the crack at $x^{*}$.

The asymptotic enrichment function $F_{\alpha }\left(r,\theta \right)$ was adopted to allow the fracture to grow and propagate. This function adds supplementary freedom degrees to the nodes related to the element containing the fracture tip using Equation (15). The symbol 𝛼 represents the node number within the element that encloses the crack tip while r and θ express the distance and the angle of the fracture within the element enclosing the fracture tip, respectively [36].

(15) $$F_{\alpha }\left(r,\theta \right)=\left\{\sqrt{r}\cos\left(\frac{\theta }{2}\right),\sqrt{r}\sin\left(\frac{\theta }{2}\right),\sqrt{r}\sin\left(\frac{\theta }{2}\right)\sin\left(\theta \right),\sqrt{r}\cos\left(\frac{\theta }{2}\right)\sin\left(\theta \right)\right\}$$

It is essential to specify the crack domain and the initial crack location within the selected domain [13]. Moreover, it is critical to define a material damage initiation criterion, such as the maximum principal strain (MAXPE) or the maximum principal stress (MAXPS) [13]. That is why the MAXPS damage criterion factor (z) was employed and can be expressed using Equation (16).

(16) $$z=\left\{\frac{\sigma_{max}}{\sigma_{max}^{o}}\right\}$$

where $\sigma_{max}^{o}$ is the maximum allowable principal stress. The ⟨⟩ symbol denotes the Macaulay bracket to indicate that pure compressive stress cannot cause or commence damage. Instead, the damage is set to start if the maximum principal stress ratio reaches 1. The maximum allowable principal stress $\sigma_{max}^{o}$ was 2.2 MPa to express the concrete tensile strength.

## 4. FE Models Verification and Discussion

**Figure 3:**
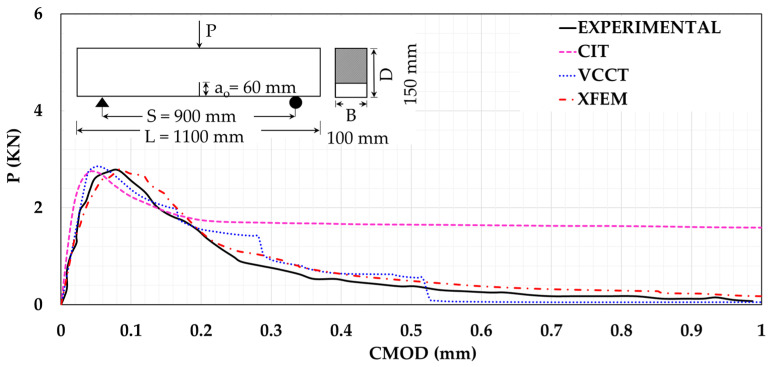
Loading level (P) versus crack mouth opening displacement (CMOD).

All models were developed using CIT, VCCT, and XFEM. Based on the results deduced from the T6 simulations, the most suitable technique was determined by using five different statistical indicators that are used by researchers in different fields [37,38,39]. These statistical indicators are: 1. root mean square error (RMSE); 2. Nash–Sutcliffe efficiency (NSE) [40]; 3. modified index of agreement (md) [41]; 4. coefficient of determination (R^2^); and 5. Kling–Gupta efficiency (KGE) [42]. The RMSE measures the differences between the experimental and the numerical data, RMSE has an optimal value of zero. The NSE determines the relative magnitude of the residual variance in the numerical data compared to the variance in the experimental data. The md estimates the additive and proportional differences in the means and variances of the experimental and numerical data. The R^2^ assesses the degree of collinearity between the numerical and experimental data. The KGE assesses the correlation, bias, and variability between the experimental and numerical data, thus providing a complete similarity estimation. The last four statistical indicators have an optimal value of one. The RMSE, NSE, md, R^2^, and KGE were computed as shown below:

(17) $$\mathrm{RMSE}=\sqrt{\frac{\sum_{i=1}^{N}({\hat{x}}_{i}-{x_{i})}^{2}}{N}}$$

(18) $$\mathrm{NSE}=1-\left[\frac{\sum_{i=1}^{N}({\hat{x}}_{i}-{x_{i})}^{2}}{\sum_{i=1}^{N}({\hat{x}}_{i}-{x^{mean})}^{2}}\right]$$

(19) $$\mathrm{md}=1-\frac{\sum_{i=1}^{N}\left|x_{i}-{\hat{x}}_{i}\right|}{\sum_{i=1}^{N}(|{\hat{x}}_{i}-x^{mean}|+\left|x_{i}-x^{mean}\right|)}$$

(20) $$R^{2}={\left(\frac{\sum_{i=1}^{N}\left[\left(x_{i}-x^{mean}\right)\left({\hat{x}}_{i}-{\hat{x}}^{mean}\right)\right]}{\sqrt{{\sum_{i=1}^{N}\left[{\hat{x}}_{i}-{\hat{x}}^{mean}\right]}^{2}}\sqrt{{\sum_{i=1}^{N}\left[x_{i}-x^{mean}\right]}^{2}}}\right)}^{2}$$

(21) $$\mathrm{KGE}=1-\sqrt{{\left(P_{c}-1\right)}^{2}+{\left(\frac{{\hat{x}}^{mean}}{x^{mean}}-1\right)}^{2}+{\left(\frac{\hat{S.D}/{\hat{x}}^{mean}}{S.D/x^{mean}}-1\right)}^{2}}$$

where N is the number of data points, $x_{i}$ is the actual experimental data value, ${\hat{x}}_{i}$ is the numerical data value, $x^{mean}$ is the experimental data mean value, ${\hat{x}}^{mean}$ is the numerical data mean value, $P_{c}$ is the Pearson’s correlation coefficient, S.D is the standard deviation of the experimental data, and $\hat{S.D}$ is the standard deviation of the numerical data. For the beam T6, the results were monitored as shown in Figure 3. From this figure, it can be deduced that, for CIT model, the flexural capacity conforms with the experimental findings. On the other hand, the post-failure stage does not reflect the experimental results. Both XFEM and VCCT were found to capture the flexural response and simulate the softening part more precisely. Table 2 shows the results of RMSE, NSE, md, R^2^, and KGE for CIT, VCCT, and XFEM and shows that both XFEM and VCCT have better correlation to the experimental results. The XFEM managed to achieve the closest optimal value for each statistical indicator, indicating that XFEM offered the best flexural simulation.

The fracture energy $\left(G_{f}\right)$ was evaluated by dividing the work ($W_{o}$) by the ligament area ($A_{L}$) of the notched beam, as given in Equation (22). From Table 3, it can be concluded that both VCCT and XFEM provide better fracture energy estimates than CIT. Moreover, XFEM gives better a estimation of the experimental result.

(22) $$G_{f}=\frac{W_{o}}{A_{L}}$$

**Figure 4:**
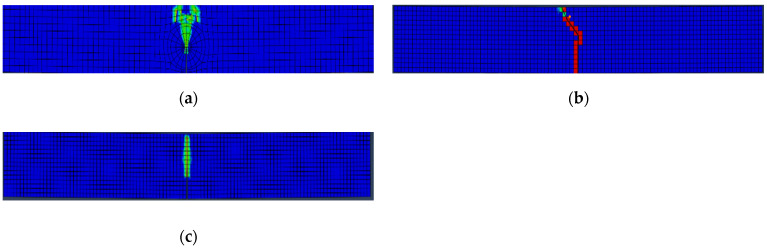
Concrete damage initiation and propagation paths; (**a**) tensile damage for contour integral technique (CIT) model; (**b**) extended finite element method (XFEM) model; (**c**) VCCT model.

According to the experimental results [14], the crack propagation path indicated the dominance of mode I fractures among all tested beam series. It can be noticed from Figure 4. That, for CIT analysis, the crack propagation path could not be captured. Instead, only a tensile damage region was captured. This is due to the stationary nature of CIT cracks. Conversely, XFEM and VCCT models showed a crack initiation and propagation path that followed the experimental behavior precisely. The initiation and propagation of concrete damage is illustrated in Figure 4.

It can be concluded that both XFEM and CIT are the most suitable and least suitable techniques for plain concrete beam fracture simulation, respectively. To support this conclusion and to identify the discrepancy between the two techniques, both XFEM and CIT were considered to verify the experimental results of the other beams stated in Table 1.

**Figure 5:**
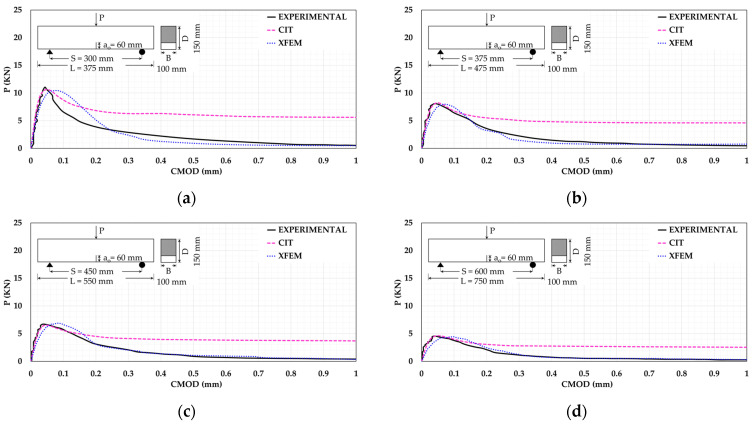

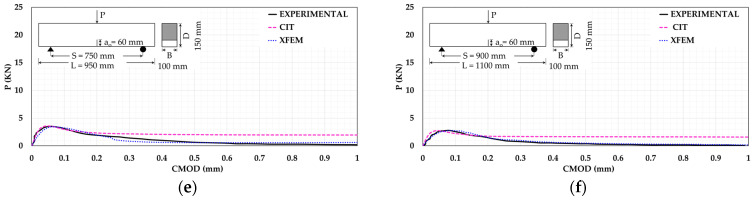
The P-CMOD relationships for beams (**a**) T2; (**b**) T2.5; (**c**) T3; (**d**) T4; (**e**) T5; (**f**) T6.

Figure 5 shows the verification of all beams using both XFEM and CIT as a relationship between P and CMOD. It can be noted that both XFEM and CIT can express the flexural capacity of all beams in a good correspondence with the experimental outcomes. As for XFEM, it can simulate the experimental flexural behavior and the post-failure softening precisely for all beams unlike CIT which shows an obvious discrepancy. These findings support the conclusion that XFEM is the most suitable approach for plain concrete beam fracture simulation.

## 5. Parametric Study and Results

In the current study, three parameters were investigated for plain and reinforced concrete beams to study their effect on the flexural behavior. These parameters were: 1. the initial notch-to-depth ratio; 2. the shear span-to-depth ratio; 3. the use of external post-tensioning. This investigation was carried out on beam T6. For reinforced concrete beams, the reinforcement consists of two top corner bars as stirrup hangers, two bottom corner bars as the main reinforcements, and shear stirrups. All bars had a diameter of 6 mm. The spacing between the stirrups was 100 mm. The reinforcement steel grade used was the standard ASTM (A 615M/A 615) grade 300. This steel grade has a minimum yield strength of 300 MPa. A constant concrete cover of 10 mm was maintained in the current work.

### 5.1. Influence of Changing Initial Notch-to-Depth Ratio

**Figure 6:**
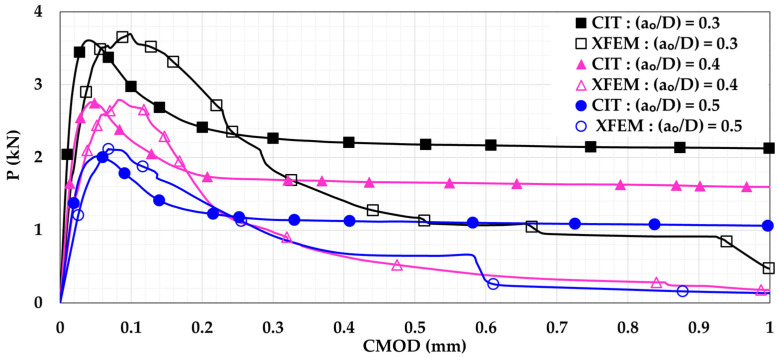
Plain concrete beam P-CMOD relationships for different initial notch-to-depth ratios (a_o_/D).

**Figure 7:**
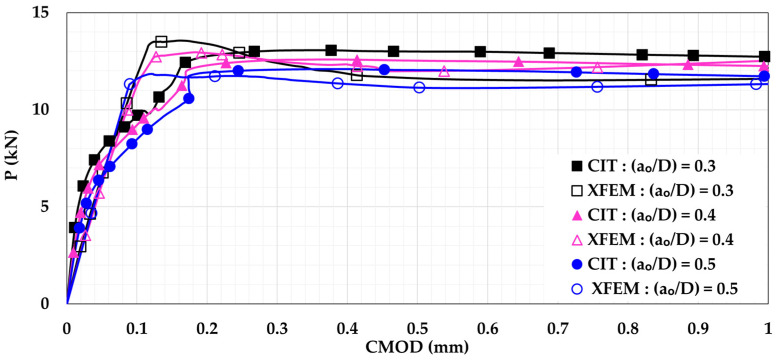
Reinforced concrete beam P-CMOD relationships for different initial notch-to-depth ratios (a_o_/D).

Initial notch-to-depth ratio (a_o_/D) values of 0.3, 0.4, and 0.5 were studied. These values imply that the corresponding values of initial notch height (a_o_) were 45 mm, 60 mm, and 75 mm, respectively. The FE models for plain and reinforced beams were executed. Figure 6 and Figure 7 show P-CMOD relationships when considering different values of a_o_/D for plain and reinforced concrete beam models, respectively. For plain concrete beams, it can be noted that the flexural capacity increases with reduction of the a_o_/D. This is because reducing the a_o_/D increases the ligament area of the notched beam, causing increased resistance to flexural damage leading to higher flexural capacity. Furthermore, a complete flexural softening can be noticed for all XFEM results unlike CIT. For the reinforced concrete beam, it can be noted that the behavior became more ductile as there was no sudden failure. Instead, a failure plateau took place. Also, the flexural capacity was drastically increased due to the presence of reinforcement. Moreover, the flexural capacity of the reinforced concrete beam seems to be nearly constant regardless the a_o_/D value. This means that even with a light amount of reinforcement, damage initiation and propagation can be controlled, causing stability. Additionally, it is noted that both the CIT and XFEM models gave very similar results, as no softening occurred in the reinforced beam. Thus, it can be concluded that CIT can give reliable results for reinforced concrete beams, unlike plain concrete beams. 

### 5.2. Influence of Changing the Shear Span-to-Depth Ratio

**Figure 8:**
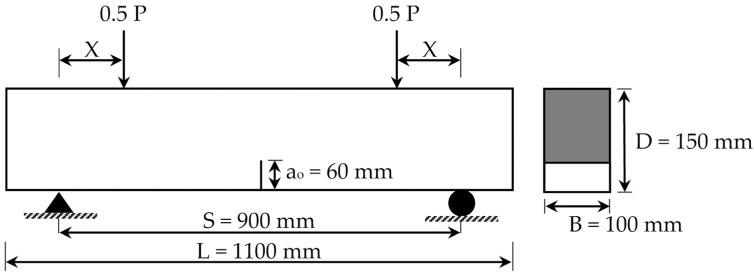
Four-point loading scheme for T6.

**Figure 9:**
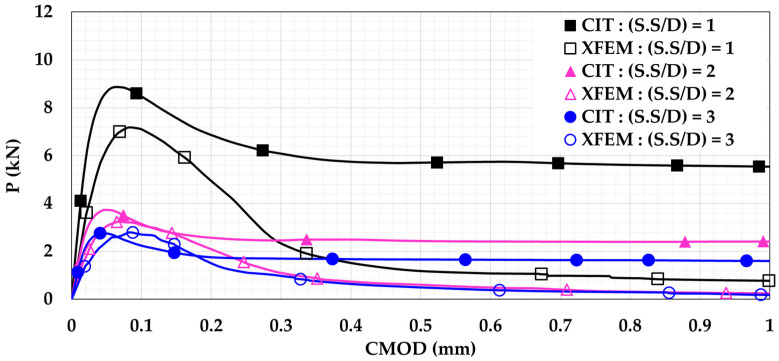
Plain concrete beam P-CMOD relationships for different shear span-to-depth ratios (S.S/D).

**Figure 10:**
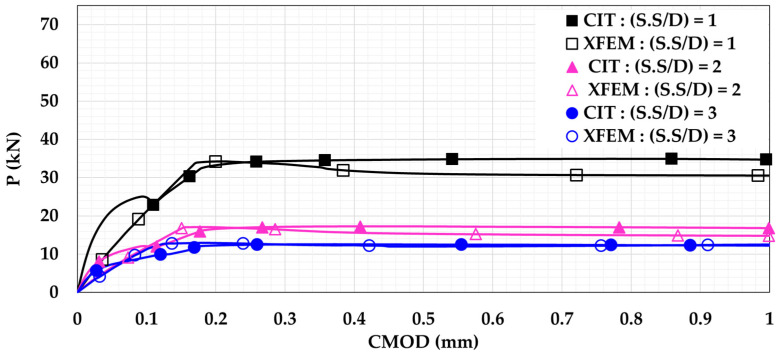
Reinforced concrete beam P-CMOD relationships for different shear span-to-depth ratios (S.S/D).

In order to study the effect of changing the shear span-to-depth ratio (S.S/D), the model was modified to follow the four-point loading setup. As seen in Figure 8, the beam was subjected to two equal loads (0.5 P). Both loads were located at the same distance (X) from the support. In the current study, the three different distances (X) considered were X = 150 mm, X = 300 mm, and X = 450 mm. These distances imply to S.S/D values of 1, 2, and 3, respectively. The P-CMOD relationships of the plain and reinforced concrete beams are shown in Figure 9 and Figure 10, respectively. These figures indicate that increasing the S.S/D decreases the flexural capacity. This is because a more direct influence is delivered to the mid-notch, causing faster crack propagation and lower flexural capacity. For plain concrete beams, XFEM showed lower flexural capacity than CIT for lower S.S/D. This means that XFEM has higher sensitivity to damage initiation and propagation than CIT. It is noted that implementing light reinforcement led to higher flexural capacity and increased ductility. It can be also noted that, after using reinforcement, the results obtained when utilizing XFEM were almost identical to the corresponding results when using CIT. This supports the conclusion that CIT is reliable for simulating reinforced concrete beams, unlike plain concrete beams.

### 5.3. Influence of Using External Post-Tensioning

**Figure 11:**
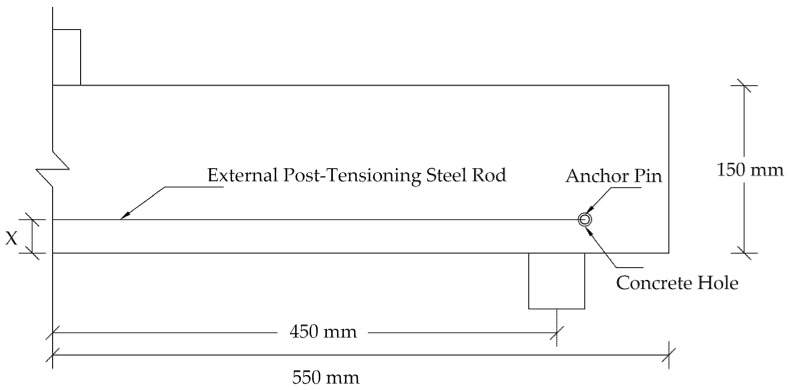
Schematic external post-tensioning (EPT) setup for beam T6.

External post-tensioning (EPT) rods were used in a three-point bending loading scheme at three locations and four levels of post-tensioning stress. This was study their effects on the flexural behavior of the plain and reinforced T6 concrete beams. These rods had a yield strength of 900 MPa [43]. The EPT configurations were different in the manner of rod location relative to the crack height, as shown in Figure 11. These locations were: 1. at the crack mouth (X= 10 mm); 2. at the middle of the crack height (X = 30 mm); and 3. at the crack tip (X = 60 mm).

Each configuration was investigated at four levels of post-tensioning stress, as follows: 1. rods are present but without post-tensioning; 2. rods are post-tensioned with 25% of their yield strength; 3. rods are post-tensioned with 50% of their yield strength; 4. rods are post-tensioned with 75% of their yield strength.

Two cylindrical steel anchor pins were placed at both ends of the concrete beam to connect the post-tensioning rods at both beam sides. Surface-to-surface contact interaction was used for the mutual surfaces between the steel anchor and the concrete beam. The motion of the steel anchor pins was constrained to the motion of the prestressing rod ends to apply the prestressing effect on the beam.

**Figure 12:**
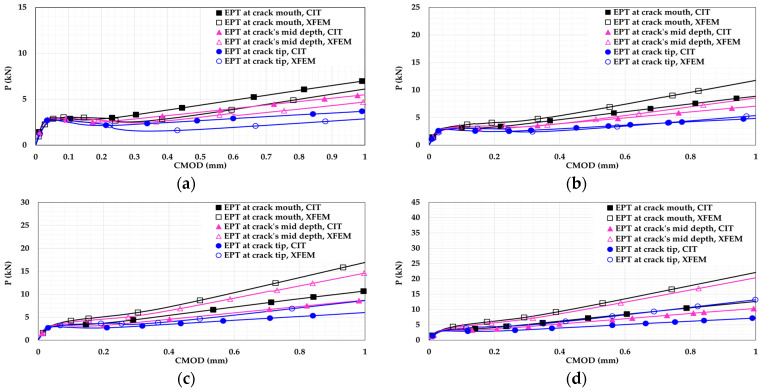
Plain concrete beam P-CMOD relationships for different EPTs at different locations: (**a**) 0%; (**b**) 25%; (**c**) 50%; (**d**) 75%.

**Figure 13:**
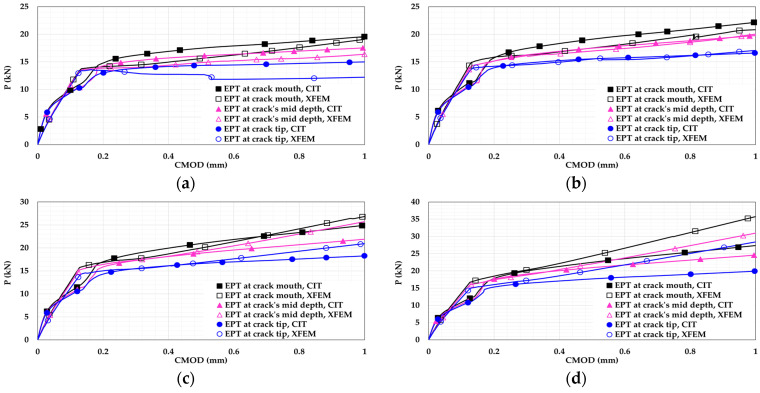
Reinforced concrete beam P-CMOD relationships for different EPTs at different locations: (**a**) 0%; (**b**) 25%; (**c**) 50%; (**d**) 75%.

For all EPT beam models, the following findings can be drawn:

1. Placing the EPT rods towards the crack mouth and increasing the EPT stress gives a better effect. This is because increasing EPT towards the crack mouth enhances the role of post-tensioning in resisting the crack opening, resulting in improved crack control.
2. For plain concrete beam models, Figure 12 shows that EPT rods increase the flexural capacity and show more ductile flexural response. The increased capacity and ductility are attributed to the contribution of post-tensioning in handling the applied stresses.
3. For plain concrete beam models, EPT rods reduce flexural softening when they are placed closer to the crack mouth. The reduction of the flexural softening occurred because the stresses were handed over to the EPT rods earlier when they were closer to the crack mouth. As result, more softening and degradation was captured for the beams having EPT rods closer to the crack tip.
4. For plain concrete beam models, increasing the EPT stress to reach 75% of the rod’s yield strength reduced flexural softening even with a rod location at the crack tip. This indicates that this amount of post-tensioning stress is capable of enhancing damage control even if the EPT rods are not located at crack mouth.
5. For plain concrete beam models, a clear discrepancy can be noticed between the CIT and XFEM results. The discrepancy is attributed to the stationary nature of a CIT crack that does not grow, preventing a complete failure regime. Conversely, XFEM is capable of representing complete damage softening.
6. For the reinforced concrete beam models, Figure 13 shows higher flexural capacity due to reinforcement presence.
7. For the reinforced concrete beam models, the flexural results are close as the reinforcement managed to control the damage initiation and propagation.
8. For the reinforced concrete beam models, both the CIT and XFEM results are in good agreement. This supports the conclusion that CIT can give trustworthy results for reinforced concrete beams, unlike plain concrete beams. Additionally, this agreement reveals that using reinforcement has more performance in controlling damage than using EPT only. This is due to the complete bond with the concrete, the bond between the reinforcement bar and the concrete is achieved along the entire bar. Conversely, EPT rods are placed outside the concrete section and are bonded to the concrete at the end anchorages only.

Based on all previous simulations, beneficial practical implications can be interpreted. For both plain and reinforced concrete fracture simulations, the XFEM model is favored over the CIT model. However, the CIT model still can be used effectively to simulate reinforced concrete fractures. Additionally, using a minimal bonded steel reinforcement is capable of controlling the fracture damage propagation and enhancing the flexural performance significantly. Moreover, decreasing shear spans to eliminate shear stress at notched sections can contribute to the escalation of the flexural capacity and fracture control performance. For rehabilitation and renovation purposes, using unbonded external post-tensioning at the crack mouth at higher stresses can improve flexural tensile capacity and tensile damage resistance.

## 6. Conclusions

This study aimed to develop numerical models using different notch modeling techniques built in ABAQUS. Notched plain and reinforced concrete beams subjected to three-point-bending and four point-bending loading setups were simulated. These notch modeling techniques were CIT, VCCT, and XFEM. The outcomes were compared with those of a pervious experimental study. Additionally, the influence of changing some parameters, such as a_o_/D, S.S/D, and EPT, was investigated. The XFEM and VCCT models simulated flexural response in good agreement with the experimental outcomes. The CIT model showed a discrepancy for plain concrete simulations but yielded reliable results for reinforced concrete beams. Furthermore, the XFEM model was considered the most suitable, as it had better fracture energy estimation, solution-dependent crack path, and lower RMSE. It was found that increasing the a_o_/D and S.S/D decreased the flexural capacity. Reinforcement implementation controlled concrete damage and increased the ductility and the flexural capacity for all studied parameters. Additionally, using EPT rods and increasing EPT stress increased the flexural capacity and ductility. Also, placing EPT rods near the crack mouth reduced flexural softening. However, for higher EPT stresses, flexural softening was reduced even if EPT rods were at the crack tip. For future research, it is recommended to extend this study by investigating the effect of using different strengthening materials to replace the bonded steel reinforcement and the unbonded steel external post-tensioning. The strengthening materials to be studied may include carbon fibers, glass fibers, and shape memory alloys.

## Figures and Tables

**Table 1:** Concrete beams studied.

Beam Series	Breadth (B) mm	Depth (D) mm	Length (L) mm	Span (S) mm	Notch Length (a_o_) mm	S/D
T2	100	150	375	300	60	2
T2.5			475	375		2.5
T3			550	450		3
T4			750	600		4
T5			950	750		5
T6			1100	900		6

**Table 2:** Results of statistical indicators.

Statistical Indicator	CIT	VCCT	XFEM	Optimal Value
RMSE	1.000	0.210	0.170	0
NSE	−3.740	0.950	0.960	1
md	0.370	0.890	0.900	1
R^2^	0.570	0.950	0.970	1
KGE	0.000	0.910	0.910	1

**Table 3:** Fracture energy $\left(G_{f}\right)$ for different analyses versus experimental results for beam T6.

	CIT	VCCT	XFEM	Experimental
$W_{o}$ (N.m)	3.380	1.175	1.260	-
$A_{L}$ (m^2^)	0.009	0.009	0.009	0.009
$G_{f}$ (N/m)	375.556	130.556	140.000	175.960

## Data Availability

The FE data used to support the findings of this study are available upon request.
